# The Synergistic Antifungal Effect and Potential Mechanism of D-Penicillamine Combined With Fluconazole Against *Candida albicans*

**DOI:** 10.3389/fmicb.2019.02853

**Published:** 2019-12-18

**Authors:** Yiman Li, Ping Jiao, Yuanyuan Li, Ying Gong, Xueqi Chen, Shujuan Sun

**Affiliations:** ^1^Department of Pharmacy, Shandong Provincial Qianfoshan Hospital, Shandong University, Jinan, China; ^2^School of Pharmaceutical Sciences, Shandong University, Jinan, China; ^3^Department of Pharmacy, Jinan Maternity and Child Care Hospital, Jinan, China; ^4^Department of Pharmacy, Wuxi People’s Hospital, Wuxi, China

**Keywords:** *Candida albicans*, fluconazole, D-penicillamine, synergy, ion homeostasis

## Abstract

Over the last few decades, candidiasis has exhibited an increasing incidence worldwide, causing high mortality in immunocompromised patients. *Candida albicans* is one of the leading opportunistic fungal pathogens. However, due to the increased use of antifungal agents, resistance of *C. albicans* to conventional agents, especially fluconazole, has frequently emerged. Therefore, research on the use of combinations of current drugs to sensitize antifungal agents and overcome fungal resistance has attracted considerable attention. This study demonstrated for the first time that D-penicillamine (PCA) combined with fluconazole showed a synergistic effect against *C. albicans*. PCA combined with fluconazole not only showed synergistic effects against planktonic cells of *C. albicans*, but also showed synergistic effects against *C. albicans* biofilms formed within 12 h *in vitro*. In addition, a *Galleria mellonella* infection model was used to evaluate the *in vivo* effects of this drug combination. The results showed that the combination of the two drugs could improve the survival rate, decrease the fungal burden, and reduce the tissue invasion of *G. mellonella* larvae. Finally, we explored the potential synergistic mechanisms of the drug combination, mainly including inhibition of the morphological transformation, reduction of the intracellular calcium concentration, and the activation of metacaspase, which is closely related to cell apoptosis. These findings might provide novel insights into antifungal drug discovery and the treatment of candidiasis caused by *C. albicans*.

## Introduction

In the last few decades, invasive fungal infections have dramatically increased in immunocompromised individuals including patients with organ transplantation and HIV infection as well as patients undergoing chemotherapy and those subjected to overprescription of steroid therapy (Romano et al., 2017). *Candida* species are the predominant fungi that are frequently present in hospital infections, and *Candida albicans* (*C. albicans*) is an important opportunistic pathogen which is the most commonly identified *Candida* species in clinical contexts. The infections caused by *C. albicans* range from superficial mucosal and dermal infections to disseminated bloodstream infections with mortality rates greater than 40% (Lohse et al., 2018). Azoles, especially fluconazole (FLC), are widely used for the prevention and treatment of infections caused by *C. albicans* due to their high efficiency, excellent bioavailability, low toxicity, and low cost. However, with the frequent use of fluconazole in clinical treatment, FLC resistant strains are easily developed, posing great challenges for clinical treatment of invasive fungal infections (Cui et al., 2015; Arendrup and Patterson, 2017; Pristov and Ghannoum, 2019). Exploration of new antifungals is time-consuming, labor-intensive, and requires a large economic investment. Therefore, combination therapy is now considered as an effective approach to improve the efficacy of antimicrobial therapy to overcome fungal resistance (Li et al., 2015). In recent years, studies have shown that many non-antifungal drugs can significantly increase the sensitivity of azoles although most of them do not have a strong antifungal effect by themselves (Liu et al., 2014). Combination therapy may be an optional approach for the treatment of invasive fungal infections, and the potential antifungal mechanisms provide new insights into novel antifungal drug development. D-penicillamine (PCA) is a thiol-containing amino acid with strong complexation of metal ions that was identified as a metabolite of penicillin (Joyce, 1989). PCA is clinically used as a first-line therapy for patients with Wilson’s disease (WD) which can cause hepatic and neurologic damages due to copper metabolism disorders. PCA is used to treat WD because of its capacity to chelate metals (Weiss et al., 2013). For the same reason, PCA has been used as an antidote for poisoning by some heavy metals. Several studies have shown that antifungal effects can be achieved by interfering with ion homeostasis in *C. albicans* (Liu et al., 2015); however, there is no report on whether PCA alone has an antifungal effect or acts synergistically with other antifungal drugs. In this study, we investigated the effects of PCA alone and in combination with FLC against *C. albicans*, and its underlying antifungal mechanisms were also explored.

In the present report, we first investigated the *in vitro* effects of PCA combined with FLC against *C. albicans*. In addition, the interactions of the drug combination *in vivo* were evaluated by establishing a *Galleria mellonella* (*G. mellonella*) larva infection model, and the molecular mechanisms of drug synergism were further investigated.

## Materials and Methods

### Strains and Growth Media

The strains used in this experiment are shown in Table 1. CA103, CA632, and CA20003 were kindly provided by Professor Changzhong Wang (School of Integrated Traditional and Western Medicine, Anhui University of Traditional Chinese Medicine, Hefei, China), and other strains were isolated from the clinical laboratory of Shandong Provincial Qianfoshan Hospital, Jinan, China. *C. albicans* ATCC10231, as a quality control strain, was kindly donated by the Institute of Pharmacology, School of Pharmacy, Shandong University, Jinan, Shandong Province, China. All strains were stored as frozen stocks of isolates in Sabouraud dextrose broth (SDB) at −80°C and were subcultured at least twice on Sabouraud dextrose agar (SDA) solid medium.

**TABLE 1:** Drug interactions of FLC and PCA against *C. albicans in vitro*.

**Strains^a^**	**MIC (μg/ml)^b^**				**FICI^b^**	**Interpretation^c^**
	**Alone**		**Combined**			
	**FLC**	**PCA**	**FLC**	**PCA**		
CA10	>512	256	0.125	64	0.25	SYN
CA16	>512	256	0.125	64	0.25	SYN
CA103	>512	512	0.125	64	0.125	SYN
CA632	>512	512	0.125	64	0.125	SYN
CA137	>512	512	0.25	32	0.06	SYN
CA20003	>512	512	1	64	0.13	SYN
CA4	1	256	0.25	32	0.375	SYN
CA8	0.5	256	0.125	64	0.5	SYN
CA19	0.25	>512	0.0625	64	0.375	SYN
CA14	0.5	>512	0.125	64	0.375	SYN
CA20	1	256	0.125	64	0.375	SYN
CA129	2	>512	0.5	16	0.28	SYN

*^*a*^CA, *Candida albicans.*^*b*^PCA, D-penicillamine; FLC, fluconazole; FICI, fractional inhibitory concentration index. MIC denotes minimum inhibitory concentration of drug that inhibited fungal growth by 80% compared with the growth control. MIC values and FICIs are shown as the median of three independent experiments. ^*c*^SYN, synergism.*

### Drugs and *Galleria mellonella* Larvae

All drugs (PCA and FLC) were purchased from Dalian Meilun Biotech Co., Ltd. (Dalian, China). The stock solutions of PCA (12,800 μg/ml) and FLC (2,560 μg/ml) were prepared in distilled water. All stock solutions were stored at −20°C before the experiment. For the *in vivo* experiment, *G. mellonella* larvae were purchased from Tianjin Huiyude Biotech Co., Ltd. (Tianjin, China). Larvae with weights ranging from 220 to 280 mg were randomly chosen and used for the experiments. Upon arrival, the larvae were stored at 12°C in the dark and subsequently used within a maximum of 2 weeks.

### Determination of Minimum Inhibitory Concentrations Against *Candida albicans* Planktonic Cells

The antifungal activities of all tested drugs against *C. albicans* were determined using the broth microdilution method in 96-well plates according to the Clinical and Laboratory Standards Institute standard M27-A3 document (CLSI, M27-A3). The strains were diluted to a final concentration of 2 × 10^3^ CFU/ml with roswell park memorial institute (RPMI) 1640 medium buffered with 3-(N-morpholino)-2-hydroxy (MOPS). The final concentrations of PCA were 8–512 μg/ml and those of FLC were 0.125–64 μg/ml. The volume of the dilution of each drug dispensed was 50 μl, yielding 100 μl per well. Each well was inoculated with 100 μl of the inoculum suspension containing 2 × 10^3^ CFU/ml. Plates were incubated at 35°C for 24 h. Growth inhibition was determined both by visual reading and by measuring the optical density at 492 nm using a microplate reader. The minimum inhibitory concentration (MIC) was defined as the concentration of drug that reduced growth by 80% (Li et al., 2017). All experiments were repeated three times. When the MIC was >512 μg/ml, the MIC value was regarded as 512 μg/ml. The *in vitro* synergistic effect was evaluated by calculating the fractional inhibitory concentration indexes (FICIs) using the following equation: FICI = FIC_*PCA*_ + FIC_*FLC*_ = (MIC of PCA in combination/MIC of PCA alone) + (MIC of FLC in combination/MIC of FLC alone). FICI values ≤0.5 are considered to indicate synergy, FICI values >4 indicate antagonism, and values between 0.5 and 4 refer to no interaction.

### Determination of Sessile Minimum Inhibitory Concentrations Against *Candida albicans* Biofilms

The sessile minimum inhibitory concentration (sMIC) of PCA combined with FLC against *C. albicans* was evaluated as described by Ramage et al. (2001) and Zhong et al. (2017) with moderate modifications. In this experiment, CA4, CA8, CA10, and CA16, four *C. albicans* strains with different FLC susceptibility levels were used to determine the sMIC of biofilms. In brief, biofilms with different maturity stages were formed by adding 200 μl of cell suspension (10^3^ CFU/ml) into 96-well flat-bottomed microtiter plates over four time intervals (4, 8, 12, and 24 h) at 35°C. At each time point, each well was carefully aspirated and gently washed three times with 200 μl of sterile phosphate-buffered saline (PBS) to remove suspended cells. Different concentrations of FLC (0.25–128 μg/ml) and PCA (16–1024 μg/ml) were added to the biofilm-coated wells. Following a further 24 h incubation at 35°C, a 2,3-bis-(2-methoxy-4-nitro-5-sulfophenyl)-2H-tetrazolium-5-carboxanilide (XTT) reduction assay was performed to examine the metabolic activity of the biofilms. Colorimetric changes in XTT reduction were measured at 492 nm using a microplate reader. The FICI model was used to illustrate the interaction between PCA and FLC against *C. albicans* biofilms. The sMIC was defined as the lowest concentration of drug that would lead to an 80% inhibition of biofilm metabolic activity compared to that of the drug-free control.

### *Galleria mellonella* Assays

To explore the synergistic effect of PCA and FLC *in vivo*, *G. mellonella* survival assays were performed according to a previously described methodology (Silva et al., 2018; Marcos-Zambrano et al., 2019). For the *G. mellonella* survival assay, *C. albicans* (CA10) was diluted in sterile PBS at a concentration of 5 × 10^8^ CFU/ml. The optimal drug concentration was screened within a clinically safe dose range of these drugs. The final concentrations of PCA and FLC were 40 and 160 μg/ml, respectively. A large number of larvae (220–280 mg) were randomly selected and divided into four groups (control, PCA, FLC, PCA + FLC) with 20 per group. A CA10 suspension (10 μl) of CA10 was injected into the last left proleg of the larvae. The larvae were kept in sterile petri dishes at 35°C for 2–2.5 h. After fungal infection, the control group was injected with 10 ml sterile PBS, and the other larvae received 10 μl of the indicated drugs as previously described (PCA, FLC, PCA + FLC) in the last right proleg. After inoculation, the larvae were placed in the dark at 35°C for observation for different time periods. The experiment was repeated three times.

The fungal burden determination was assessed by CFU counts at different stages after inoculation for 4 days (Krezdorn et al., 2014). The larvae were pretreated as described above, but each group included 30 larvae. During inoculation, five larvae were randomly selected from each group on each day and placed in a pre-sterilized tube with 5 ml of sterile PBS. After homogenization, the homogenate was diluted with PBS, and 10 μl of the homogenate at each dilution was collected and spread on solid yeast extract peptone dextrose (YPD) medium. Colony counts were performed after incubation for 24 h at 35°C. The experiment was repeated three times.

### Hyphae Formation

Morphological transformation of *C. albicans* occurred in RPMI1640, and a hypha-inducing effect was observed (Chang et al., 2012; Liu et al., 2017). The CA10 strain (2 × 10^5^ CFU/ml) was incubated in a 96-well plate. Morphological transitions resulting from different drug interventions were examined according to groups as follows: 1 μg/ml FLC, 64 μg/ml PCA, 1 μg/ml FLC + 64 μg/ml PCA and control group. The 96-well plate was placed in a constant temperature shaking incubator (75 rpm, 35°C) for 4 h. Then, the cell suspensions were aspirated, and each well was washed with 200 μl PBS to remove the non-adherent cells. At the indicated time, the cells were observed and photographed by an Olympus fluorescence microscope (Leica DMi8, Germany). The experiment was repeated three times.

### Intracellular Calcium Detection

Calcium homeostasis is important for the normal growth of *C. albicans* and is closely related to its pathogenicity (Gao et al., 2014; Liu et al., 2015). Fluorescent probe Fluo-3/AM (3 mM) was used to determine fluctuations of intracellular calcium. The intracellular fluorescence intensity was measured as described previously (Shi et al., 2010). In brief, CA10 cells were activated, harvested, and washed three times by D Hank’s buffer solution (calcium ion, magnesium ion, and phenol red free, Solarbio, China). Then cells were suspended at a concentration of 10^7^ CFU/ml and stained with 5 μM Fluo-3/AM for 45 min at 37°C in the dark. Unstained Fluo-3/AM was removed by washing with the same buffer. Then drugs of different groups (1 μg/ml FLC, 64 μg/ml PCA, 1 μg/ml FLC + 64 μg/ml PCA and control) were added. The formula used to calculate the concentration of calcium was as follows: [Ca^2+^]_*i*_ = *K*_*d*_ × (*F* − *F*_*min*_)/(*F*_*max*_ − *F*). *K*_*d*_ is the effective dissociation constant of calcium binding to Fluo-3/AM (204 nmol/L at 37°C). *F* is the mean fluorescence intensity (MFI) for each sample. *F*_*min*_ is the background fluorescence intensity with 0.1% Triton X-100 and 0.1 mM EDTA solution, and *F*_*max*_ is the maximum fluorescence intensity with 0.1% Triton X-100 plus 1 mM CaCl_2_ solution. Calcium levels were determined by a flow cytometer (Becton Dickinson FACSARIAII, United States) at an excitation wavelength of 488 nm and an emission wavelength of 525 nm. Changes in calcium concentration were measured every 10 min for 50 min. The experiment was repeated three times.

### Metacaspase Activation

Caspase is a class of cysteine-aspartic proteases that, when activated, cleave proteins to transmit proteolytic cascade signals, triggering apoptosis (Dai et al., 2012). Although caspases are not present in fungi, orthologs of the caspase family, termed metacaspases, have been identified in *C. albicans* (Uren et al., 2000). FITC-VAD-FMK *in situ* Marker (Sigma) was used to visualize metacaspase activation in *C. albicans*. Observation of the fluorescence intensity was carried out as described above (Wu et al., 2010; Lee and Lee, 2014; Yun and Lee, 2016). Four groups were used in the experiment, including the control, FLC (0.5 μg/ml), PCA (128 μg/ml), and FLC (0.5 μg/ml) + PCA (128 μg/ml). CA10 suspensions 0 (5 × 10^6^ CFU/ml) were treated with different drugs for 12 h at 35°C. Then, the cells were washed twice with PBS, suspended at a volume of 500 μl with PBS containing 10 μM FITC-VAD-FMK *in situ* Marker and incubated for 30 min in the dark at 35°C. Then, 30 μl of the cell suspension was placed on a glass slide, and the fluorescence was visualized using a fluorescence microscope (Leica DMi8, Germany). The experiment was repeated three times.

### Uptake and Efflux of Rhodamine 6G

The uptake and efflux of rhodamine 6G (Rh6G) in *C. albicans* CA10 cells were examined according to a previously described protocol (Jiang et al., 2013; Li et al., 2015, 2016). The cell suspension was adjusted to 1 × 10^5^ CFU/ml and incubated in YPD liquid medium for 18–19 h at 35°C. Next, the cells were collected and washed with glucose-free PBS three times and resuspended at a concentration of 5 × 10^6^ CFU/ml. Then, the cells were shaken for 1 h in a shaking incubator (200 rpm) at 35°C in PBS (without glucose) to fully deplete the energy of the cells. The Rh6G uptake assay was conducted as follows: Rh6G (10 μM) and PCA (64 μg/ml) were added to the cells mentioned above at the same time, and the PCA-free group was considered as the control group. The MFI of intracellular Rh6G was assessed by flow cytometry (Becton Dickinson FACSARIAII, United States) at an excitation wavelength of 488 nm and an emission wavelength of 530 nm which was measured every 10 min for 50 min. The Rh6G efflux assay was conducted as follows: 10 mM Rh6G was added to the de-energized cells, and the cells were incubated for 1 h. Then, the cells were washed and resuspended with PBS (5% glucose). The final concentration of PCA was 64 μg/ml, and Rh6G alone was considered as the control group. The MFI of intracellular Rh6G was measured every 30 min for 150 min and the protocol was the same as that used in the Rh6G assay mentioned above. The experiment was repeated three times.

### Statistics

All experiments were independently performed at least three times. Graphs were generated and statistical analyses were performed with GraphPad Prism 7 (GraphPad, La Jolla, CA, United States) and IBM SPSS Statistics 22 (SPSS, Chicago, IL, United States). The fungal burden, intracellular calcium concentration, and rhodamine 6G uptake and efflux were analyzed using an unpaired *t*-test. A *P*-value of <0.05 was considered significant.

## Results

### The Minimum Inhibitory Concentrations Against Planktonic Cells of *Candida albicans*

The data in Table 1 show that the antifungal effect of PCA alone against *C. albicans* was not obvious, with an MIC >256 μg/ml. However, when PCA and FLC were combined against planktonic cells of *C. albicans*, the MIC of FLC was significantly reduced, indicating a strong synergistic effect. Among the six FLC-resistant strains, PCA could reduce the MIC of FLC from >512 μg/ml to 0.125–1 μg/ml, and the FICI value ranged from 0.06 to 0.25. When used in combination with FLC against FLC-sensitive *C. albicans*, PCA could reduce the MIC of FLC from 0.25–2 to 0.0625–0.5 μg/ml, and the FICI value was 0.28–0.5. Based on the FICI values, we demonstrate that PCA exhibits *in vitro* synergistic antifungal activity with FLC against planktonic cells of *C. albicans*.

### The sMICs Against *Candida albicans* Biofilms

Based on the synergistic effect of PCA combined with FLC *in vitro* against planktonic cells of *C. albicans*, the activity of this drug combination against pre-formed biofilms of *C. albicans* was further studied with four representative strains. As exhibited in Table 2 PCA could significantly reduce the sMIC of FLC against 4, 8, and 12 h biofilms with an FICI < 0.5, showing strong synergism between PCA and FLC against the four tested strains. However, the FLCI value >0.5 for biofilms with a pre-formation time of 24 h indicated no synergistic effect.

**TABLE 2:** *In vitro* interactions of PCA with FLC against biofilms of *C. albicans*.

**Strains^a^**	**Time (h)^b^**	**sMIC (μg/ml)^c^**				**FICI^c^**	**Interpretation^d^**
		**Alone**		**Combined**			
		**PCA**	**FLC**	**PCA**	**FLC**		
CA4	4	>512	512	32	0.25	0.0625	SNY
	8	>512	>1,024	64	1	0.064	SNY
	12	>512	>1,024	64	1	0.064	SNY
	24	>512	>1,024	>1,024	>128	>0.5	NI
CA8	4	512	>1,024	64	0.25	0.125	SYN
	8	512	>1,024	64	0.5	0.125	SYN
	12	512	>1,024	128	2	0.25	SYN
	24	>512	>1,024	>512	>256	>0.5	NI
CA10	4	>512	512	128	1	0.25	SNY
	8	>512	>1,024	128	1	0.25	SNY
	12	>512	>1,024	512	4	0.51	SNY
	24	>512	>1,024	>1,024	>128	>0.5	NI
CA16	4	>512	>1,024	64	1	0.126	SYN
	8	512	>1,024	64	1	0.126	SYN
	12	1,024	>1,024	256	1	0.25	SYN
	24	>512	>1,024	>512	>1,024	>0.5	NI

*^*a*^CA, *Candida albicans.*^*b*^Time indicates incubation period of preformed biofilms. ^*c*^PCA, D-penicillamine; FLC, fluconazole; FICI, fractional inhibitory concentration index. sMIC denotes sessile minimum inhibitory concentration of drug that produced an 80% reduction of biofilms metabolic activity compared with the growth control. sMIC values and FICIs are shown as the median of three independent experiments. ^*d*^SYN, synergism; NI, no interaction.*

### *Galleria mellonella* Assays

**FIGURE 1:**
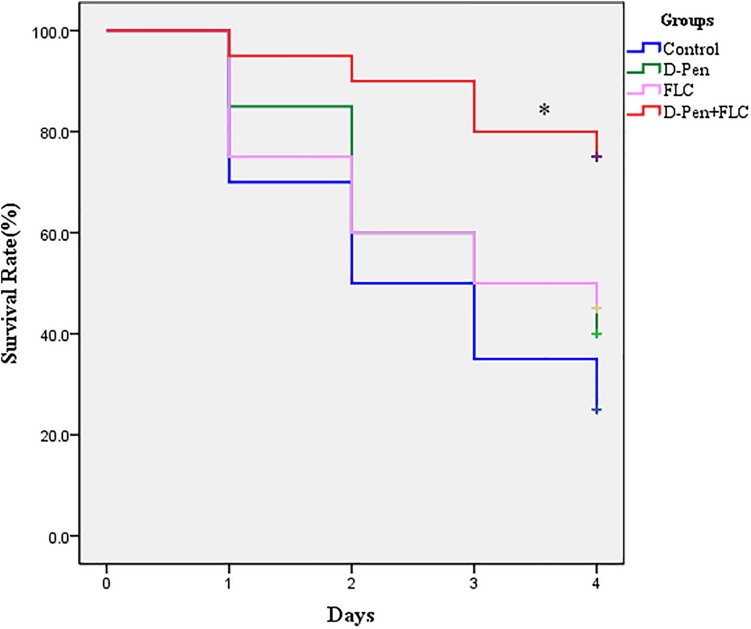
Survival curve of *Galleria mellonella* infected with *Candida albicans*. The concentration of yeast cells was 5 × 10^8^ CFU/larva. Treatments consisted of PBS, fluconazole (FLC) (160 μg/ml), D-penicillamine (PCA) (40 μg/ml), and a combination of FLC (160 μg/ml) with PCA (40 μg/ml). The data came from the means of three independent experiments. ^∗^*P* < 0.05.

For the *G. mellonella* survival assay, larvae were incubated at 35°C for up to 4 days post-infection, and larval death was recorded daily by visual inspection. Figure 1 shows no significant difference among the control, PCA alone, and FLC alone groups (*P* > 0.05). Larvae infected with CA10 and treated with a combination of PCA (40 μg/ml) and FLC (160 μg/ml) were protected from infection when compared with other groups (*P* < 0.05). Therefore, these data suggest that the drug combination protects *G. mellonella* larvae from *C. albicans*’ induced mortality.

**FIGURE 2:**
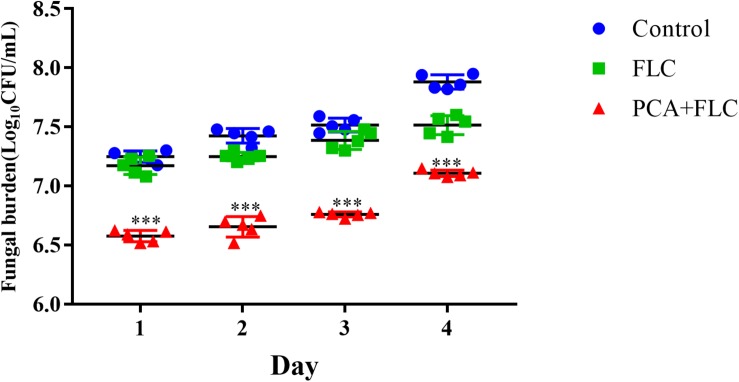
Fungal burden of *Galleria mellonella* infected with *Candida albicans.* The concentration of yeast cells was 5 × 10^8^ CFU/larva. Treatments consisted of PBS, fluconazole (FLC) (160 μg/ml), PCA (40 μg/ml), and a combination of FLC (160 μg/ml) with PCA (40 μg/ml). Data came from the means of three independent experiments. For clarity, data for treatment with PCA are not shown because the data obtained closely followed those shown for the control group. The data came from the means of three independent experiments. ^∗∗∗^*P* < 0.001.

To explore the combined effect of PCA and FLC on the fungal burden of the infected larvae, a fungal burden analysis was performed. Since the results of the PCA group were very similar to those of the control group, the PCA group data were removed to make the results more concise and clearer. As can be seen, Figure 2 shows no significant effect on the fungal burden in the larvae infected with CA10 in the FLC group compared to the control group (*P* > 0.05). However, the fungal burden of the two-drug combination group was significantly lower (*P* < 0.001), indicating that the combination of PCA and FLC could reduce the amount of fungal burden in the larvae infected with *C. albicans*.

### Hyphae Formation

**FIGURE 3:**
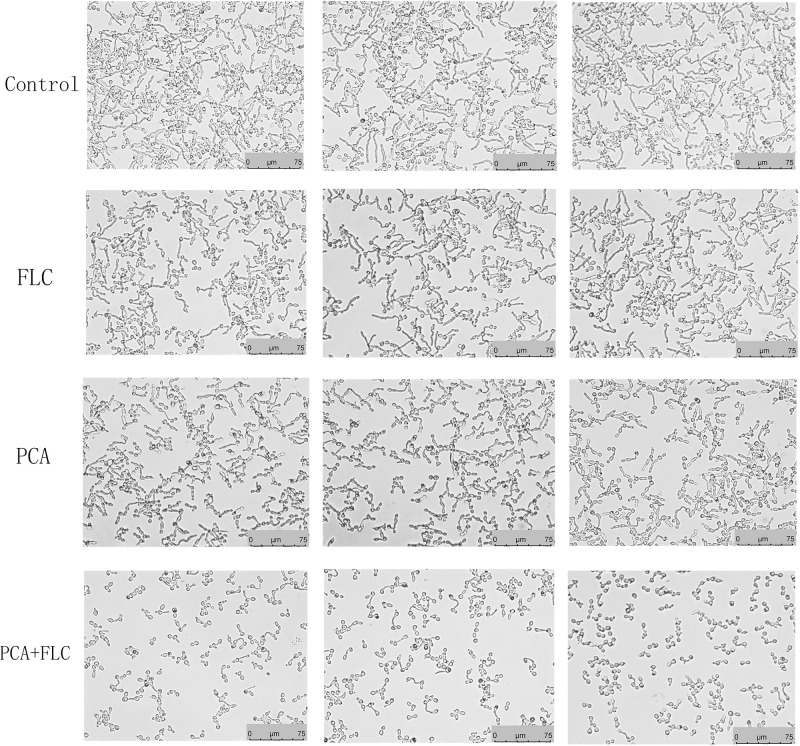
Effect of D-penicillamine (PCA) on *Candida albicans* hyphal growth. PCA and fluconazole (FLC) were diluted in hyphae-inducing media, RPMI 1640 medium, at a final concentration of 64 and 1 μg/ml, respectively. The cellular morphology was photographed after incubation at 37°C for 4 h. The photographs were collected from three independent experiments.

Hyphae formation, as a virulent determinant, plays a vital role in the pathogenesis of *C. albicans*. RPMI 1640 is a kind of medium capable of inducing hyphae formation. As displayed in Figure 3, *C. albicans* formed dense and long hyphae in RPMI 1640 in the control group. Almost no difference was observed when comparing the PCA and FLC single-use groups to the control group. In contrast to the above three groups, the drug combination group had the shortest hyphal length, and the amount of hyphae was also significantly reduced. The drug combination of PCA and FLC could inhibit the filamentous growth of CA10, resulting in reduced drug resistance and virulence of *C. albicans.*

### Intracellular Calcium Detection

**FIGURE 4:**
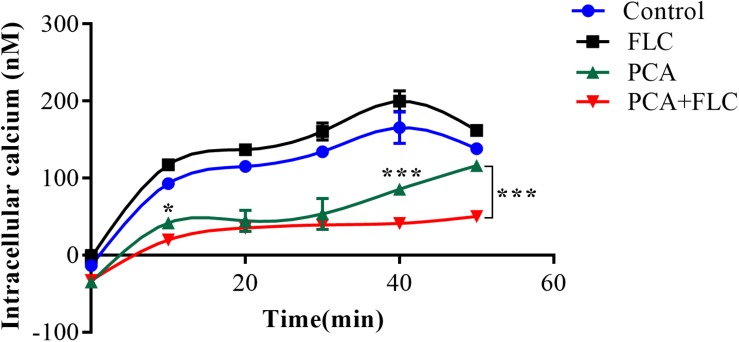
Fluctuation of intracellular calcium concentration in *Candida albicans.* CA10 cells with Fluo-3/AM staining were analyzed by the flow cytometry after treatments with different drugs (1 μg/ml fluconazole (FLC), 64 μg/ml D-penicillamine (PCA), 1 μg/ml FLC + 64 μg/ml PCA and control). The data came from the means of three independent experiments. ^∗^*P* < 0.05, ^∗∗∗^*P* < 0.001.

The calcium concentration upon treatment with a combination of PCA and FLC was determined by the fluorescent calcium indicator Fluo-3/AM, and the results for CA10 are shown in Figure 4. We measured the changes in intracellular calcium concentration every 10 min during the first 50 min. As shown in Figure 4 the trend was similar in the control group and the fluconazole group. In both groups, calcium increased slowly in the first 40 min and slowly decreased in the next 10 min. The PCA group showed a slight change in the first 30 min and increased rapidly after 30 min. The changes in the drug combination group were more stable, and calcium obviously decreased at 40 and 50 min compared with the other three groups (*P* < 0.001), indicating that the fluctuation of calcium may be a potential antifungal mechanism for the actions of the combination of PCA and FLC against *C. albicans*.

### Caspase Activation

**FIGURE 5:**
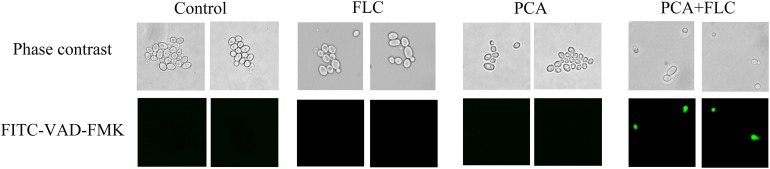
Effect of D-penicillamine (PCA) on the activity of metacaspase in *Candida albicans*. CA10 cells with FITC-VAD-FMK staining were observed under a fluorescent microscope after treatments with fluconazole (FLC) (0.5 μg/ml), PCA (128 μg/ml), and a combination of FLC (0.5 μg/ml) with PCA (128 μg/ml). The photographs were collected from three independent experiments.

The results are shown in Figure 5. There were almost no fluorescent cells in the control group and the groups with single drug application. Moreover, many cells were visible under the microscope. However, in the PCA and FLC combination groups, the number of cells was significantly reduced, and the cells emitted bright green fluorescence, indicating activation of intracellular metacaspases. The results demonstrated that metacaspase, as a central regulator of apoptosis, was associated with the fungal cell death induced by the drug combination.

### Uptake and Efflux of Rhodamine 6G

**FIGURE 6:**
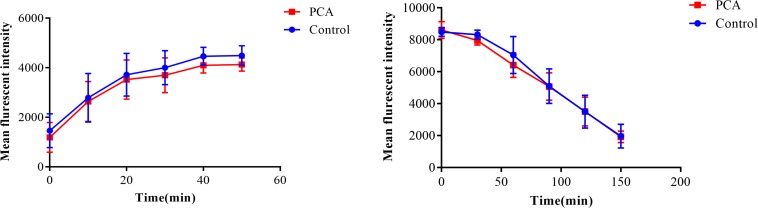
Effect of D-penicillamine (PCA) on the uptake and efflux of rhodamine 6G (Rh6G) in *Candida albicans.* The uptake and efflux of Rh6G in the absence and presence of PCA (64 μg/ml) were determined by a flow cytometer. Mean fluorescence intensity represent the intracellular Rh6G in *C. albicans*. The data came from the means of three independent experiments.

As shown in Figure 6, there was no obvious difference between the PCA group and the control group regardless of the uptake and efflux of Rh6G (*P* > 0.05), indicating that the synergy between PCA and FLC was not related to the uptake and efflux of FLC.

## Discussion

Although the isolation rate of non-*Candida albicans* fungi is increasing, *C. albicans* is still a major opportunistic fungus capable of causing a broad spectrum of diseases. Azoles and echinocandins are the most common drugs used to treat *Candida* infections. However, the implementation of antifungal drugs cannot keep pace with the increased incidence of drug resistance. To resolve this emerging problem, alternative synergistic drug combinations have been explored among antifungals and non-antifungals resulting in a new direction in antifungal drug discovery and antifungal therapy. In recent years, many studies have reported that some compounds with no antifungal effects can enhance the activity of antifungal drugs (Shi et al., 2010; Jiang et al., 2013; Li et al., 2016; Lu et al., 2018). Our study concluded that the combination of PCA and FLC exhibited effective antifungal effects against *C. albicans* both *in vitro* and *in vivo*, which may have implications for the prevention of drug resistance. Ions exhibit important physiological functions in fungi. In addition, studies have confirmed that ion homeostasis is closely associated with antifungal drug resistance, fungal virulence, cell wall integrity, morphological transformation, and oxidative stress responses in *C. albicans*. It is worth mentioning that the calcium-mediated signaling pathway is closely associated with the pathogenicity, virulence, and drug resistance of invasive fungal strains. Therefore, disturbing ion homeostasis in *C. albicans* may be an effective method to combat invasive fungal infections (Cyert and Philpott, 2013; Malavia et al., 2017; Gerwien et al., 2018; Li et al., 2018).

D-penicillamine is used clinically to treat WD for its prominent role in chelating metal ions. In this study, we tested the antifungal effect of PCA when used alone or in combination. Among the six FLC-resistant strains, PCA could reduce the MIC of FLC from >512to 0.125–1 μg/ml. When used in combination with FLC against FLC-sensitive *C. albicans*, PCA could reduce the MIC of FLC from 0.25–2 μg/ml to 0.0625–0.5 μg/ml. The results demonstrated that PCA combined with FLC showed synergistic effect against *C. abicans* The FICI was used to determine the interaction between the two drugs using the checkerboard method, and the FICI value of the PCA-FLC combination was ≤0.5, indicating that PCA and FLC synergistically inhibited the planktonic cells of *C. albicans*.

Biofilm is defined as a structured community of microorganisms embedded within a self-produced matrix consisting of extra polymeric substances (EPS). Biofilms represent one of the major virulence factors contributing to the pathogenesis of candidiasis. Moreover, cells encased in biofilm matrix exhibit increased resistance to antifungal agents (Rasmussen and Givskov, 2006). Unlike planktonic organisms, cells encased in a biofilm matrix build a heterogeneous and natural drug-tolerant environment (de Mello et al., 2017). *C. albicans* can form biofilms on medically implanted devices in hospitalized patients, resulting in great challenges in clinical treatment. In this study, we demonstrated that the combination of PCA and FLC had a strong synergistic effect on biofilms that had been formed for 4, 8, and 12 h, and the sMIC of FLC decreased from >1024 to 1–4 μg/ml, indicating that the drug combination exerts broad prospects for prevention or early treatment of biofilm-related infections.

Insects are currently widely employed to evaluate the virulence of fungal pathogens and to assess the *in vivo* efficacy and toxicity of antimicrobial drugs. The *in vivo* activity of PCA–FLC combination was assessed by a *G. mellonella* infection model due to its low cost and ease of use. The doses of FLC and PCA were converted and explored according to the doses considered safe for human infections. The survival rate assay revealed the primary evaluation of the *in vivo* effect. Figure 1 shows that the survival rate in the group treated with the combination of PCA and FLC was two times greater than that of fluconazole alone, and three times greater than that of the control group, indicating that the combined treatment significantly increased the survival rate of *G. mellonella*. The fungal burden observed in *G. mellonella* larvae corresponded to the survival rate experiment. The results of the fungal burden experiment showed that the combination of the two drugs could significantly reduce the amount of *C. albicans* remaining in the larvae. Figure 2 shows that although the CFU in the drug combination group gradually increased during the post-infection period, the rate of increase was much slower than that of the control group and the FLC monotherapy group, indicating that the drug combination could effectively clear *C. albicans* cells in larvae. In general, the combination of PCA and FLC significantly enhanced the antifungal efficacy *in vivo*, which was consistent with the *in vitro* effects.

To investigate the molecular mechanism of drug synergism, we conducted the following explorations. We first measured the effects of the drug combination on yeast-to-hyphae transformation. *C. albicans* has the ability to switch its morphological form from unicellular budding yeast to a hyphae form. It is generally accepted that the yeast form is non-virulent, while the hyphae form possesses greater virulence (Leng et al., 2001). Filamentation represents an important virulence factor in the pathogenesis of *C. albicans*, therefore inhibiting the transformation from yeast to hyphae is an effective method to combat candidiasis (Wilson et al., 2016). Figure 3 shows that after 4 h of incubation, the filamentation in the PCA-FLC group was much shorter and looser than that of the other three groups. The results revealed that drug combination therapy could significantly inhibit the yeast-hyphae transition of *C. albicans*.

In recent years, the calcium signaling pathway has received extensive attention due to its essential role in fungal survival. Calcium, acting as a secondary messenger molecule, participates in a wide range of cellular processes in both fungi and mammals. Calcium homeostasis is essential in many cell biological processes and is critical for cell growth. Moreover, the calcium signaling pathway has been a focus of extensive studies due to its potential roles in the stress response, morphogenesis, biofilm formation, virulence, resistance, and pathogenicity of *C. albicans* (Gao et al., 2014). Previous studies demonstrated that doxycycline and calcium channel blockers combined with FLC could produce a synergistic effect against *C. albicans*, and its mechanism seemed to be associated with calcium disturbance (Shi et al., 2010; Gao et al., 2014). As shown in Figure 4, compared to the other three groups, we observed that the calcium of the PCA and FLC combination groups was the lowest during the measurement period. Moreover, the calcium was significantly decreased at 10, 40, and 50 min compared to the PCA group (*P* < 0.05).

Two types of apoptosis occur in yeast, including caspase-dependent and caspase-independent apoptosis. Yeast caspase-dependent apoptosis accounts for 40% of the investigated cell death scenarios (Madeo et al., 2009). Caspase belongs to a family of cysteine protease peptidases that include a cysteine residue as the catalytic nucleophile. Caspase can specifically cleave certain proteins, leading to the occurrence of apoptosis (Shirazi and Kontoyiannis, 2015). Metacaspase is a the homologous form of caspase that exists in *C. albicans* and has actions similar to caspase in mammalian cells. FITC-VAD-FMK is a fluorescent dye that is used to monitor the caspase response due to its ability to penetrate the cell membrane, its low toxicity and its ability to be irreversibly activated by caspase (Shirazi and Kontoyiannis, 2013). As shown in Figure 5, there were no fluorescent cells in the control and monotherapy groups. However, the cells treated with PCA + FLC exhibited with bright green fluorescence, and the number of cells was significantly decreased compared to the other three groups. The results suggested that the drug combination might activate the activity of metacaspase in *C. albicans*.

It is widely acknowledged that overexpression of drug efflux pumps is one of the most common mechanisms of resistance in *C. albicans*. Increased expression of the efflux transport pumps results in an improved ability of the cells to pump out the drugs, resulting in a decreased effective concentration of intracellular antifungal drugs. This study showed that PCA combined with FLC had no effect on the drug efflux pumps (*P* > 0.05), suggesting that the synergistic effect of the drug combination might be independent of drug efflux pumps.

## Conclusion

D-penicillamine had synergistic effects with FLC against not only planktonic cells but also biofilms of both sensitive and resistant *C. albicans* strains. Moreover, PCA plus FLC increased the survival rate of *G. mellonella* larvae infected with *C. albicans* as well as decreased the fungal burden. Mechanism studies elucidated that the synergism is related to inhibition of the morphological transformation, disruption of intracellular calcium homeostasis, and activation of metacaspase. This study could provide new insights for fighting against drug resistance and developing novel antifungal drugs.

## Data Availability Statement

All datasets generated for this study are included in the article/supplementary material.

## Author Contributions

YML and SS conceived and designed the experiments and wrote the manuscript. YML performed the experiments. All authors analyzed the data, contributed the reagents, materials, and analysis tools, and approved the manuscript for publication.

## Conflict of Interest

The authors declare that the research was conducted in the absence of any commercial or financial relationships that could be construed as a potential conflict of interest.
